# Challenges in critical care services in Sub-Saharan Africa: Perspectives from Nigeria

**DOI:** 10.4103/0972-5229.53112

**Published:** 2009

**Authors:** U. V. Okafor

**Affiliations:** **From:** Department of Anesthesia, University of Nigeria teaching hospital, Ituku Ozalla, Enugu, Nigeria

**Keywords:** Africa, critical care challenges, Nigeria, Sub-Saharan

## Abstract

Critical care services in Nigeria and other West African countries had been hampered by economic reversals resulting in low wages, manpower flight overseas, government apathy towards funding of hospitals, and endemic corruption. Since then things have somewhat improved with the government's willingness to invest more in healthcare, and clampdown on resource diversion in some countries like Nigeria. Due to the health needs of these countries, including funding and preventive medicine, it may take a long time to reach reasonably high standards. Things are better than they were several years ago and that gives cause for optimism, especially with the debt cancellation by Western nations for most countries in the region. Since most of the earlier studies have been done by visiting doctors, mainly outside the West African subregion, this paper seeks to present a view of the challenges faced by providers of critical care services in the region, so that people do not have to rely on anecdotal evidence for future references.

## Introduction

It has been reported that modern intensive or critical care medicine emerged in the 1950s, largely pioneered by the anesthetist, Dr. Bjorn Ibsen during the polio epidemic at the Kommune hospital in Copenhagen in 1953.[1] This area developed following advances in mechanical ventilation and cardiopulmonary resuscitation.[2,3] In most developing countries, especially those of Sub-Saharan Africa, intensive care medicine or critical care services are poorly developed, or at most, still in infancy. Special intensive care units (ICUs), like neurological and neonatal ICUs, are still a novel concept.

The challenges faced during the development of critical care services in the region are enormous.[4,5] Most of the work on the challenges in critical care services in Sub-Saharan Africa, except South Africa, has been done by expatriate authors on short-term service and have mainly focused on regions outside West Africa, which ironically is one of the most populous regions of Africa.[6,7]

These challenges include inadequate manpower, especially consultant anesthetists who run ICUs in the subregion.[8,9] This was partly due to the fact that the specialty was not popular among medical graduates, who had their eyes on specializing in areas that would enable them to operate private clinics in order to supplement their then meager salaries.

The resulting increase in salary since the return of civilian rule in Nigeria in 1999 coincided with an increase in the number of doctors entering the specialty of intensive care with all specialists receiving the same salary scale.[8]

This shows that as often stated, the problem with healthcare delivery in most parts of the region has much to do with the political will of the government and its priorities (which does not often include human investment). Unlike most Sub-Saharan African countries, medical practice in Nigeria is mainly doctor-based even in rural areas, though this spread is in no way nationwide.

The first ICU in Nigeria was established at the University of Nigeria Teaching Hospital (UNTH), Enugu, in 1973, following the successful management of cardiac surgery patients there.[10] Subsequently, other Federal Universities/tertiary care centers developed their own ICUs.

Critical care nursing was introduced in the country in 1982 by the Department of Anesthesia of the Jos University Teaching Hospital (JUTH), Nigeria.[11] Currently, there are two hospitals that train critical care nurses in Nigeria. Their national association is a member of the World Federation of Critical Care Nurses (WFCCN), having been admitted in 2007. Currently, the Nigerian association has about 380 members. It is not clear if every critical care nurse is registered with the association.[11] However, the number is small for a nation of more than 140 million (figures vary) people. So, it can be safely assumed that there are not enough critical care nurses to work in the ICUs, making for an average of about ten critical care nurses for each of the nation's 36 states.

Evolution of critical care services in Nigeria can be divided into phases based on the nation's economic resources. The oil boom era (from early 1970s to the mid-1980s) was a period of growth and development with employment of expatriate staff and overseas-trained Nigerians. Following the economic reversal from the mid-1980s to late 1990s, critical care services basically fell into disrepair, with serious regression, inadequate material and manpower, low morale, and manpower flight overseas. The increase in oil revenues coupled with political will has gradually improved the delivery of critical care services since the turn of this century, even if the number of specialist anesthetists remains grossly inadequate.[4,8]

Due to this paucity of anesthetists, some tertiary care centers in the country do not have ICUs, even when there are funds to operate a few bedded ICU.

Another area of manpower limitation is the paucity of biomedical engineers and even anesthetic technicians. This has led to a poor maintenance culture which hinders critical care services in the region. There is also the need to train healthcare personnel on how to use the procured modern equipments, as there are reports of materials not being used due to a lack of knowledge on how to use these equipments.[5] This can be achieved by organizing workshops with hands-on experience. Unlike some other African countries, Nigeria has not been a major beneficiary of philanthropic or religious organizations who have helped greatly in other parts of the continent. These organizations provide manpower and materials that are, in some cases, of first-world standard in both practice and training of health personnel. Some Nigerians have been beneficiaries of their expertise, but are mainly self-sponsored.

During the early years of medical postgraduate training in Nigeria, each trainee specialist spent a year in Western Europe, usually the United Kingdom, updating both knowledge and skills, before the reversal of economic fortunes led to its suspension. There is a need for that program to be revived, so as to help doctors from Nigeria to keep pace with the rapid advances in medicine. The Overseas Doctor's Training Scheme (ODTS) by the Royal Colleges in the UK can help by offering limited training posts to doctors from Sub-Saharan Africa willing to return to their countries to help train others. Exchange programs with other nations like South Africa and the Indian subcontinent with advanced healthcare systems and more hands-on experience should be encouraged.

Unlike reports from some African studies regarding availability of therapies like oxygen,[12,13] it is relatively cheap in Nigeria, oxygen being locally manufactured. This has made oxygen concentrators, which are expensive, unnecessary. Piped medical gases and vacuum are used in most teaching hospitals.

Drugs for use in the ICU including potent parenteral antibiotics and inotropes, are generally available under generic names. Parental nutrition which was a standard practice two decades ago when I was a house officer is rarely used nowadays due to the massive de-evaluation of the local currency since that period, which has made it beyond reach for most patients.

Blood products like fresh frozen plasma and clotting factors are rarely available. This is somewhat surprising since hematological services are fairly well developed with well-trained practitioners. There is another side to this story as scoring systems like The Acute Physiological And Chronic Health Evaluation (APACHE) cannot be used routinely due to the high cost of the required laboratory investigations.

Invasive monitoring, which is the standard practice in modern critical care, is rarely used due to the high cost of the material needed.

The daily cost of an ICU bed is quite expensive in some centers (about USD 85 in our center). Since hospital bills are borne by patients in most cases, cost effectiveness and cost benefit are a major consideration in admitting patients. Though there is a National Health Insurance Scheme (NHIS) in the country, it is mainly restricted to government and large company employees.

As reported in some studies from Africa, the main groups benefiting from ICU care are postoperative surgical patients.[14–17] Our center which is Nigeria's top hospital for cardiac surgery also has a separate ICU for open heart surgery patients. Predominant medical diagnoses in our ICU were cerebrovascular accidents, myasthenia gravis, Guillian Barre' syndrome,[9] and cardiomyopathies. Tetanus, reportedly a major player in some African ICUs,[6,18] is very rare in our ICU, similar to another Nigerian study.[19] This may be due to the effective immunization drives with special focus on rural areas.

Erratic water and power supply can be considered a problem in Nigeria, but not in a few other West African countries like Ghana and Cote'd' Voire, with more stable power supply. It remains a major problem in Nigeria despite the government's efforts to improve the situation. These auxiliary services need to be developed in tandem with improved quality of critical care services.

The abundance of disposable latex gloves and sharp object disposal bins has resulted in a decreased risk of staff infection.

Intensive care unit mortality rates vary according to the study population. In a study of severe head-injury patients in the ICU of an elite national hospital in the federal capital territory of Abuja, Nigeria, the mortality rate was 68.4%.[20] In another study of medical neurological and obstetric patients admitted to our ICU in Enugu, the mortality rates were 43.5[9] and 33%, respectively.[21] In critical care obstetric patients admitted to the ICU of the University College Hospital, Ibadan, Nigeria, the mortality rate was 52%,[22] lower than another obstetric group study from Burkina Faso (60%).[23]

Mortality rates in the general ICU population in two studies from Nigeria and Uganda were 35.1[19] and 25%,[6] respectively.

Corruption is believed by many to have adversely affected health care delivery in Nigeria, and conversely, critical care services. It is believed that if money budgeted for equipment is properly used, the improvement would be massive. The recent upgrading of most federal government funded teaching hospitals in Nigeria to international standard portends well for the future as internal audits have already shown some improvement in patient care. Future studies are needed to validate that trend.
